# Transgenic expression of non-structural genes of Theiler’s virus suppresses initial viral replication and pathogenesis of demyelination

**DOI:** 10.1186/s12974-016-0597-4

**Published:** 2016-06-01

**Authors:** Hyun Seok Kang, Jinjong Myoung, Eui Young So, Young Yil Bahk, Byung S. Kim

**Affiliations:** Department of Microbiology-Immunology, Northwestern University Medical School, 303 East Chicago Ave., Chicago, IL 60611 USA; Present address: Korea Zoonosis Research Institute, Chonbuk National University, Chollabuk-Do, 570-390 Republic of Korea; Present address: Department of Orthopaedics, Warren Alpert-Medical School, Brown University-Rhode Island, Providence, RI USA; Present address: Department of Biotechnology, Konkuk University, Chungju, Chunbuk 380-701 Republic of Korea

**Keywords:** TMEV, P2/P3 transgenic mice, T cells, CNS, Pathogenesis

## Abstract

**Background:**

Chronic infection with Theiler’s murine encephalomyelitis virus (TMEV) in susceptible SJL/J mice induces an immune-mediated demyelinating disease and has extensively been used as a relevant infectious model for multiple sclerosis (MS). Infection of the host with many other viruses also leads to acute or chronic inflammatory diseases in the central nervous system (CNS). Levels of viral load in the host often play a critical role in the pathogenesis of virus-induced diseases. Thus, the inhibition of viral replication in the host against a broad spectrum of similar viruses is critically important for preventing the viral pathogenicity.

**Methods:**

P2/P3-expressing transgenic (B6 X SJL)F1 founders were generated and bred onto the C57BL/6 and SJL/J backgrounds. Differences in the development of demyelinating disease were compared. Viral persistence, cytokine production, and immune responses in the CNS of infected control and P2/P3-Tg mice were analyzed after infection using quantitative PCR, ELISA, and flow cytometry. Various cell types from the control and P2/P3-Tg mice, as well as cells transfected in vitro with the P2 and/or P3 regions, were also analyzed for viral replication and innate cytokine production.

**Results:**

P2/P3-transgenic (P2/P3-Tg) mice carrying the viral non-structural protein genes displayed significantly reduced virus-specific T cell responses in the CNS against both the structural and non-structural proteins. Consequently, viral loads in the CNS were greater in the Tg mice during the chronic infection. However, P2/P3-Tg SJL mice exhibited reduced disease incidence and less severe clinical symptoms than did their non-transgenic littermates. Interestingly, P2/P3-Tg mice showed low viral loads in the CNS at a very early period after infection (1–3 days) with TMEV and related EMCV but not unrelated VSV. Cells from P2/P3-Tg mice and cells transfected with the P2 and/or P3 regions in vitro yielded also lower viral replication but higher IFN-α/β production.

**Conclusions:**

This study demonstrates that the expression of viral non-structural genes in mice inhibits initial viral replication and suppresses sustaining pathogenic anti-viral immune responses to broad viral determinants. It appears that the elevation of innate immune cytokines produced in the cells expressing the non-structural viral genes upon viral infection is responsible for the inhibitions. The inhibition is partially virus-specific as it is more efficient for a related virus compared to an unrelated virus, suggesting a role for the similarity in the viral genome structures. Therefore, the expression of viral non-structural genes may serve as a useful new method to prevent a broadly virus-specific pathogenesis in the hosts.

## Background

Theiler’s murine encephalomyelitis virus (TMEV)-induced demyelinating disease has been extensively studied as a relevant animal model for human multiple sclerosis (MS). Intracerebrally infected susceptible SJL/J mice develop neuropathological symptoms that exhibit many immunological and pathological similarities to MS and thus serve as an infectious model for this immune-mediated disease [1, 2]. Immune responses have been implicated in the development of TMEV-induced demyelinating disease [3–5], although the relative importance of virus-specific over autoimmune responses to disease development remains unclear. The majority of immune responses to TMEV are against the capsid proteins encoded by the P1 region in the TMEV-induced demyelinating disease [6–8]. We previously showed that SJL/J mice expressing the TMEV P1 region mounted lower levels of viral epitope-specific CD4^+^ and CD8^+^ T cell responses throughout the course of viral infection and developed less severe disease [8]. Lower levels of virus-specific T cell responses in P1-transgenic SJL/J mice led to higher viral persistence compared to their littermates. However, the level of disease development was also dampened due to the reduced number of pathogenic T cells in the transgenic mice. In contrast to the abundant immunological studies on the P1 proteins, very few studies have investigated the effects of the P2 and P3 regions (non-structural proteins).

We previously demonstrated that the majority CD4^+^ T cell responses following TMEV infection in susceptible SJL mice but not in resistant C57BL/6 mice targeted the 3D polymerase encoded by P3 [9]. This result was consistent with an early study that indicated that transgenic expression of the TMEV P2/P3 region did not alter the resistance of C57BL/10 mice (H-2^b^) to TMEV-induced disease [10]. However, their subsequent studies with transgenic mice expressing TMEV 3D in the susceptible FVB background demonstrated resistance to the pathogenesis of demyelinating disease [11, 12]. Therefore, it is conceivable that 3D-transgenic FVB mice may be self-tolerant to the induction of potentially pathogenic T cell responses. Investigations into the potential contributions of P2/P3-specific immune responses to the development of TMEV-IDD will be valuable for elucidating the potential roles of the TMEV P2 and P3 regions in the pathogenesis of demyelinating disease.

To investigate the effects of the P2/P3 genes in the development of TMEV-IDD, we generated transgenic mice expressing the P2 and P3 regions of TMEV under the control of the hCMV promoter. P2/P3-Tg mice in the resistant B6 background cleared the viral load similar to the control B6 mice. In contrast, P2/P3-Tg mice in the susceptible SJL/J background displayed higher viral loads in the central nervous system (CNS) but less pathogenesis. T cell responses to the P1 (structural capsid) and P2/P3 (non-structural protein) epitopes were significantly reduced in the P2/P3-Tg mice regardless of their backgrounds. Thus, the reduction in both protective and pathogenic T cell responses may cause such increased viral loads but decreased pathogenesis. Interestingly, macrophages and astrocytes from P2/P3-Tg mice infected with TMEV in vitro produced reduced levels of IL-6 and viral messages compared to the cells from naïve control mice. Additionally, NIH-3T3 cells transfected with P2, P3, or P2/P3 showed marked inhibition of viral particle production accompanied by an increase in the type I IFN response compared to untransfected or control plasmid-transfected cells. The inhibition of viral replication in cells from P2/P3-Tg mice appeared to be somewhat virus-specific because the replication of TMEV and closely related encephalomyocarditis virus (EMCV) was inhibited, whereas the inhibition of the replication of unrelated vascular stomatitis virus (VSV) was transient. These results strongly suggest that transgenic expression of non-structural viral genes may be able to induce innate immunity, which inhibits the initial replication of related viruses. Thus, these results open the possibility that gene expression of a part of the viral genome could be used to prevent the virally induced chronic disease caused by related viruses.

## Methods

### Animals

P2/P3-expressing transgenic (B6 X SJL)F1 founders were generated at the transgenic core facility of Northwestern University. A transgenic founder F1 mouse was bred onto the C57BL/6 and SJL/J backgrounds for more than eight generations in a specific pathogen-free facility prior to use. The breeder C57BL/6 and SJL mice were purchased from Harlen Laboratories (Indianapolis, IN, USA). All experiments were conducted with 6- to 10-week-old females. Experiments using animals were conducted according to the permission (#2011-1316 for Byung Kim) of the Animal Care and Use Committee at Northwestern University.

### Synthetic peptides and antibodies

All peptides were purchased from GeneMed (GeneMed Synthesis Inc, South San Francisco, CA, USA) and used as described previously [13]. All antibodies were purchased from BD Pharmingen (San Diego, CA, USA).

### Viruses and cell lines

The BeAn strain of TMEV used in this study was propagated and titered in BHK-21 cells grown in Dulbecco’s modified Eagle medium supplemented with 7.5 % donor calf serum. For intracerebral (i.c.) infection, 30 μl of virus solution containing 3 × 10^6^ PFU was injected into the right cerebral hemisphere of 6–8-week-old mice anesthetized with isoflurane. Clinical symptoms of disease were assessed weekly using the following grading scale: grade 0 = no clinical signs; grade 1 = mild waddling gait or flaccid tail; grade 2 = severe waddling gait; grade 3 = moderate hind limb paresis; and grade 4 = severe hind limb paralysis.

### Reverse-transcription PCR

Total cellular RNA was isolated from various tissues of P2/3 transgenic mice, including brain, spinal cord, spleen, thymus, liver, and kidney, using the Trizol^®^ reagent (Invitrogen, CA, USA). First-strand complementary DNA (cDNA) was synthesized from 1 μg of total RNA by utilizing the SuperScript^®^ III First-Strand Synthesis Supermix (Invitrogen, CA, USA) at 55 °C. The relative concentrations of cDNA were equalized among the groups based on the level of glyceraldehyde-3-phosphate dehydrogenase (GAPDH) amplification (25 cycles) by polymerase chain reaction (PCR). Primers for the control GAPDH gene and P2/P3 transgene were purchased from Integrated DNA Technologies: GAPDH, 5′-AACTTTGGCATTGTGGAAGG-3′ and 5′-ACACATTGGGGGTAGGAACA-3′ and the P2/P3 transgene (3D), 5′-CTGCAATTGGAACTGACCCAGATG-3′ and 5′-ATGTCGTGACACAG-CCAGAGAT-3′.

### Plaque assay

Virus titers in the infected CNS tissues were enumerated using a standard plaque assay on BHK-21 cell monolayers [14]. To visualize the plaques, the monolayer was stained using 0.1 % crystal violet after fixation with methanol.

### Isolation of CNS-infiltrating MNC

Mice were perfused with sterile Hank’s balanced salt solution (HBSS), and the excised brains and spinal cords were homogenized. CNS-infiltrating mononuclear cells (MNCs) were enriched in the 1/3 bottom fraction of a continuous 100 % Percoll (Pharmacia, Piscataway, NJ, USA) gradient after centrifugation for 30 min at 27,000×*g*, as described previously [15].

### Intracellular staining of cytokine production

Freshly isolated CNS-infiltrating MNCs were cultured in 96-well round bottom plates in the presence of relevant or control peptides as described previously [16]. Allophycocyanin-conjugated anti-CD8 (clone Ly2) or anti-CD4 (clone L3T4) antibodies and a PE-labeled rat monoclonal anti-IFN-γ (XMG1.2) antibody were used for intracellular cytokine staining. Cells were analyzed on a Becton Dickinson FACS Calibur or FACS Sort flow cytometer. Live cells were gated based on light scatter properties.

### IFN-γ and IL-17 ELISA

Mouse IFN-γ and IL-17 ELISA kits were purchased from BD Biosciences (San Diego, CA, USA) and R&D Systems, Inc. (Minneapolis, MN, USA), respectively. Cytokine levels in splenic culture supernatants were assessed according to the manufacturer’s instructions. Briefly, diluted samples were incubated for 2 h with plate-bound capture antibodies after blocking for 1 h. Cytokine expression levels were visualized using HRP-conjugated detection antibodies in the presence of the HRP substrate TMB (BioFX Laboratories, Owings Mills, MD, USA). The absorbance was measured at 450 nm.

### T cell proliferation assay

The proliferative activity of epitope-specific CD4^+^ T cells was measured based on [^3^H]thymidine incorporation levels. Spleen cells from P2/P3 transgenic and control mice 7 dpi were stimulated with PBS, the CD4 peptide mix, or plate-bound anti-CD3/CD28 antibodies for 3 days, then pulsed with 1 μCi [^3^H]thymidine-deoxyribose for 18 h. Cells were harvested and ^3^H incorporation was measured using TopCount. Data are expressed as CPM ± SD of triplicates.

### Generation of H-2K^s^ tetramers

H-2K^s^ tetramers were generated as previously described [17]. Briefly, H-2K^s^ and the human β2-microglobulin gene were subcloned into the pET28 bacterial expression vector. BL21/DE3 competent cells were transformed and protein expression was induced with IPTG for 4–5 h. Inclusion bodies were purified and refolded in the presence of peptides. The soluble monomeric form of the peptide-MHC complex was biotinylated with BirA at room temperature and tetramerized with streptavidin-PE (Invitrogen, Carlsbad, CA, USA).

### Virus replication assay

Plasmids (pMIG) containing the non-structural genes P2, P3, or P2/P3 were generated after inserting the corresponding genes cloned from pSBW. Phoenix cells were transfected with Lipofectamine-2000 (Invitrogen, Carlsbad, CA, USA) to generate retroviruses. NIH-3T3 cells were infected with the recombinant retroviruses at room temperature for 90 min. After 48 h, eGFP-positive cells in the defined gate were sorted using the FACSAria flow cytometer (BD Biosciences, San Diego, CA, USA). After 2 weeks of rest, the cells were infected with TMEV at a multiplicity of infection (MOI) of 10 for 6, 12, 18, and 24 h. Supernatants were collected at each time point for plaque assays. For some experiments, real-time PCR was used to determine gene expression levels. The lowest expression level was set as onefold expression.

### Statistical analysis

Data are shown as the mean ± SD of two to three independent experiments or triplicates of one representative experiment from at least three independent experiments. The significance of differences in the mean values was determined by Student’s *t* test. *P* values < 0.05 were considered statistically significant.

## Results

### P2/P3 transgene is expressed in various organs of Tg mice

**Fig. 1:**
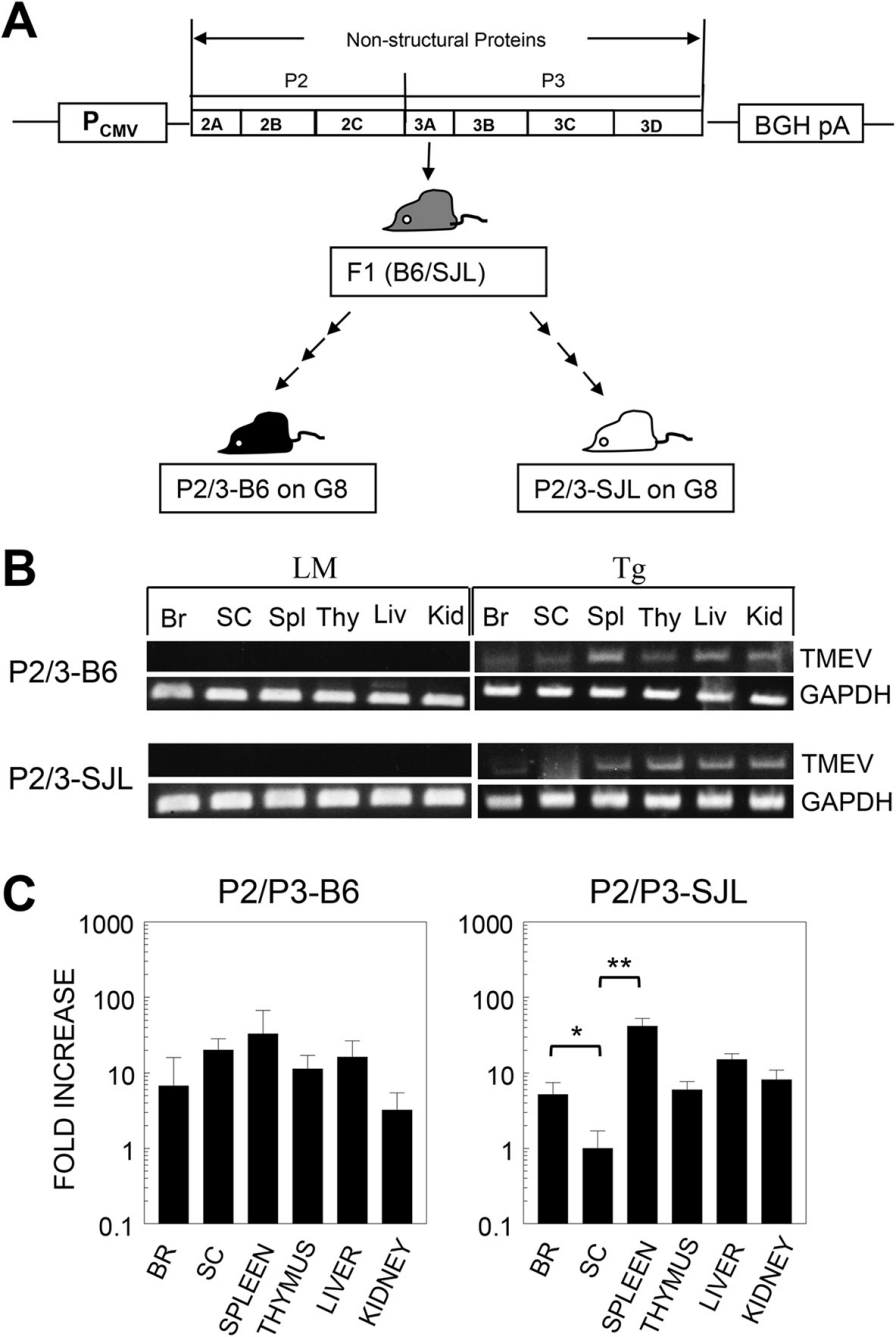
Expression of viral P2/P3 transgenes in multiple organs. **a** Schematic diagram of the P2/P3 construct controlled by the CMV promoter. BGH pA represents the bovine growth hormone polyadenylation signal. (C57BL/6 X SJL)F1 founder mice were backcrossed to either B6 or SJL mice more than eight times. **b** The expression of the 3D transgene region in the brain (*Br*), spinal cord (*SC*), spleen (*Spl*), thymus (*Thy*), liver (*Liv*), and kidney (*Kid*) was examined by RT-PCR. Littermates (*LM*) negative for the transgene resulting from the backcrossing were used as the control mice. Amplification of the GAPDH gene was compared with the viral gene expression in the samples. Representative RT-PCRs of Tg lines in the C57BL/6 and SJL backgrounds are shown. **c** Relative levels of transgene expression in various organs were compared using semi-quantitative real-time PCR. DNase pre-treated total RNA was used to synthesize the cDNA. The statistical significance of the differences was analyzed by Student’s *t* test. *P < 0.05; **P < 0.01

To induce “immune unresponsiveness” to the P2/P3 region gene products, we generated P2/P3 Tg mice with P2/P3 driven by the human CMV promoter similar to the previously described P1 Tg mice using the BeAn strain of TMEV [8]. A transgenic (B6 X SJL)F1 founder expressing the P2/P3 region of the TMEV genome was bred in the SJL and B6 backgrounds for at least eight generations (G8) prior to use (Fig. 1a). To analyze the expression of the viral P2/P3 genes in the Tg mice, cDNA was prepared from various organs (brain, spinal cord, spleen, thymus, and kidney). The 3D polymerase region in the cDNA was amplified by conventional PCR. Transgene expression was detectable in all of the organs of the Tg mice but not in the organs of their littermates (LM) (Fig. 1b). Semi-quantitative real-time PCR was performed to determine the relative levels of the transgenes in multiple organs (Fig. 1c). The levels of Tg expression differed up to 10-fold among the various organs from Tg mice in the B6 background, although these expression differences were not statistically significant. In contrast, Tg mice in the SJL background showed 10- to 100-fold differences among the organs. Interestingly, transgene expression was particularly low in the spinal cords of the SJL Tg mice. However, viral protein expression in these organs was not detectable by Western blotting and ELISA using polyclonal antibodies to the N-terminal peptides of P2 region (not shown). Therefore, the level of viral proteins produced in the Tg mice seems to be low.

### P2/P3-Tg SJL mice display reduced incidence and severity of TMEV-induced demyelinating disease compared with SJL mice

**Fig. 2:**
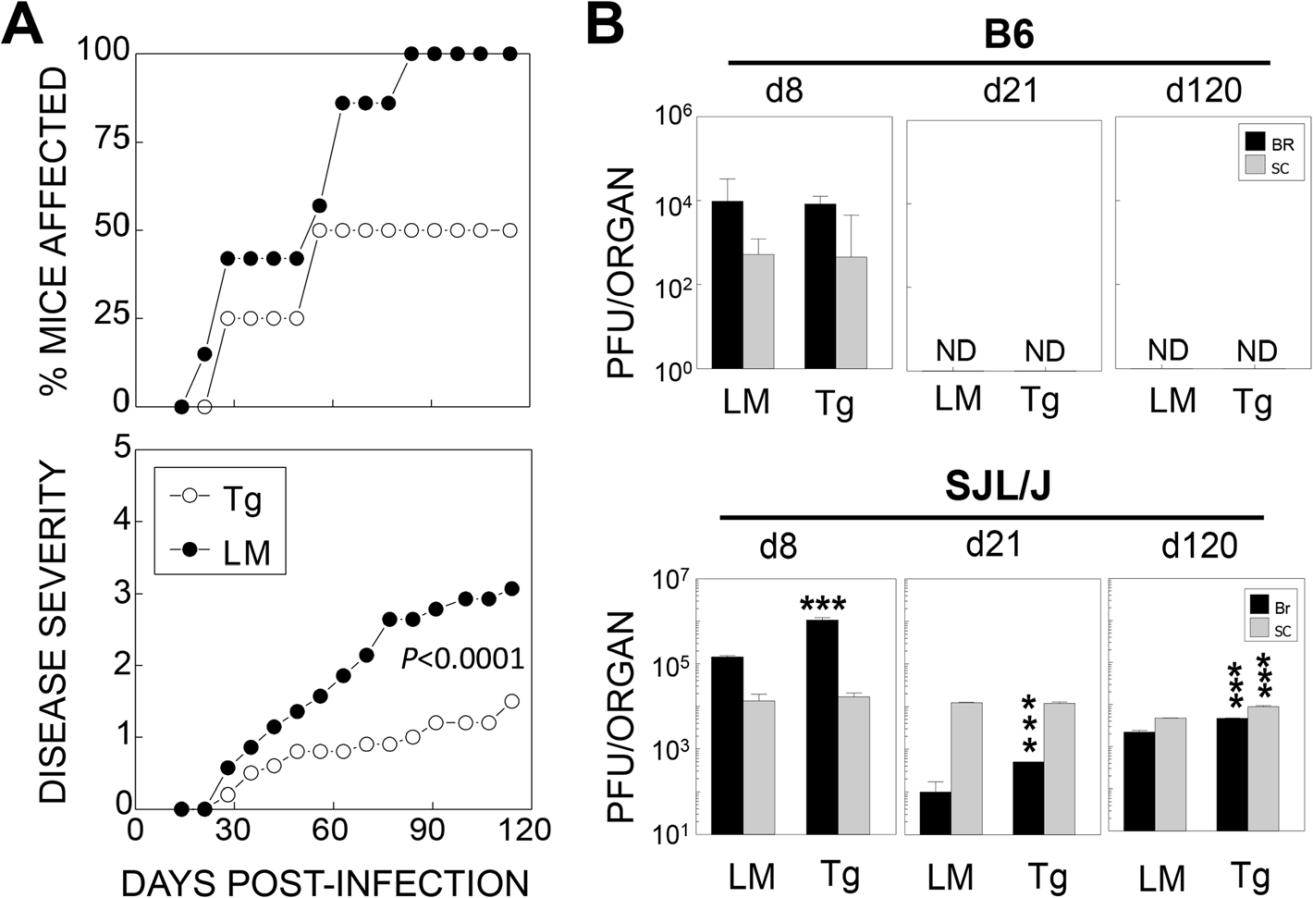
Reduced disease severity but elevated viral loads in TMEV-infected P2/P3-Tg mice in the SJL background. **a** Development of clinical disease in TMEV-infected P2/3-Tg mice (*n* = 15) and their littermates (LM, *n* = 15) in the SJL background was examined for 120 days after infection with TMEV. Clinical symptoms were assessed weekly based on behavioral changes as described in the “Methods” section. No clinical symptoms were developed in the P2/P3-Tg mice in the B6 background (data not shown). **b** The titers of the infectious virus in the CNS of TMEV-infected P2/P3-Tg mice and their littermates were determined by plaque assay on the indicated number of days post-infection. The PFU numbers represent the PFUs per whole brain or spinal cord calculated from the pool of three to four mice per group. The statistical significance of the differences was analyzed by Student’s *t* test between control and Tg mice. *P < 0.05; **P < 0.01; and ***P < 0.001. d8, d21, and d120 represent day 8, day 21, and day 120 post-infection, respectively

To investigate the impact of P2/P3 expression on virus persistence and the disease course, we infected P2/P3-Tg mice and their littermates with TMEV (Fig. 2). During the 120-day disease course, both the P2/P3-Tg B6 mice and their littermates remained disease free (data not shown). However, the P2/P3-Tg SJL mice and their littermates developed clinical signs consistent with the TMEV-induced demyelinating disease (Fig. 2a). It has been extensively documented that the TMEV-induced clinical symptoms faithfully correlate with the cellular infiltration and demyelination in the white matter, including TMEV-infected P1-Tg and 3D-Tg mice under the susceptible backgrounds [8, 12]. Interestingly, the P2/P3-Tg SJL mice showed reduced disease incidence and severity compared to their control littermate SJL mice. We also assessed viral loads in the CNS for correlation with disease development. P2/P3-Tg B6 mice and their B6 littermates cleared the viral infection by day 21 post-infection (Fig. 2a). In contrast, both the P2/P3-Tg SJL mice and their littermates failed to clear their viral loads (Fig. 2b). Interestingly, P2/P3-Tg SJL mice with higher viral loads in the CNS developed less severe disease and exhibited a reduced disease incidence (Fig. 2a, c). These results are consistent with previous observations [8] indicating that the level of viral persistence is not correlated with the development of TMEV-IDD and viral persistence itself does not cause disease development.

### Both P1 and P2/P3 epitope-specific CD4^+^ T cell responses are reduced in P2/P3-Tg mice compared their control littermates

**Fig. 3:**
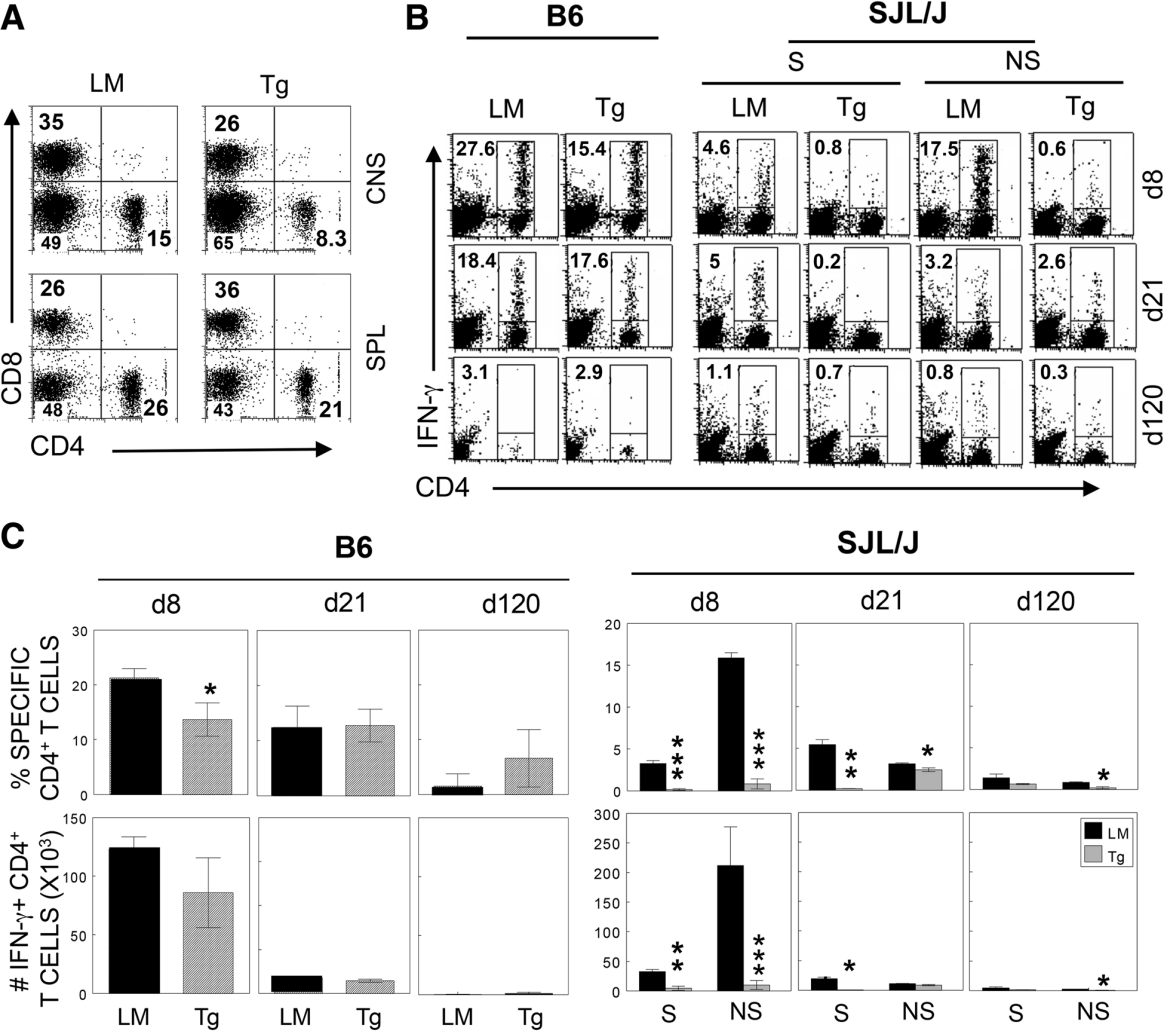
Levels of antigen-specific IFN-γ-producing CD4^+^ T cells in the CNS of TMEV-infected P2/P3-Tg mice (Tg) and their littermates (LM). **a** Splenocytes and CNS-infiltrating MNCs isolated from TMEV-infected mice were stained with CD4 and CD8. **b** CNS-infiltrating MNCs were restimulated with the mixture of CD4^+^ T cell-specific epitopes (mix for cells from B6 mice, VP2_206–220_, and VP4_25–38_; S mix for cells from SJL mice, VP1_233–250_, VP2_74–86_, and VP3_24–37_; NS mix, 3D_6–23_, and 3D_20–38_). After 6 h of stimulation, the cells were stained for CD4 and intracellular IFN-γ. The percentages of CD4^+^ and IFN-γ^+^ cells are shown in the upper left corner. Data are representative of three independent experiments. **c** Histograms represent the mean percentages (*upper panels*) and the numbers (*lower panels*) from three independent experiments assessed by flow cytometric analysis. Statistical significance was analyzed by Student’s *t* test. *P < 0.05; **P < 0.01; and ***P < 0.001

It was expected that the P2/P3-Tg mice would induce comparable T cell responses against the TMEV structural protein (P1) upon infection, although these Tg mice might display immune tolerance to the P2/P3 epitopes due to neonatal exposure to the antigens. To test this possibility, we analyzed CNS-infiltrating T cells from the infected mice (Fig. 3). The proportions of both CD4^+^ and CD8^+^ T cells were lower in the P2/P3-Tg B6 (Fig. 3a) and SJL (not shown) mice compared to their control littermates. To examine whether the reduced proportions of infiltrating T cells reflected a decrease in antigen-specific T cell responses, we assessed the proportions and numbers of CNS-infiltrating virus-specific CD4^+^ T cells during the course of infection in the P2/P3-Tg and control mice (Fig. 3b, c). In the P2/P3-Tg B6 mice, the proportion and number of IFN-γ-producing CD4^+^ T cells were much lower at early time points (8 dpi), although they became similar at 21 dpi (Fig. 3b, c). In contrast, virus-specific IFN-γ-producing CD4^+^ T cell responses to both the non-structural (*P* = 0.0008) and structural epitopes (*P* = 0.00003) in the P2/P3-Tg SJL mice were severely compromised compared to their littermates (Fig. 3b, c). These data suggest that the transgenic expression of the P2/P3 genes induces a pan-tolerance of CD4^+^ T cell responses to TMEV epitopes rather than tolerance limited to the P2/P3 epitopes.

### CD8^+^ T cell responses to TMEV were significantly reduced in P2/P3-Tg mice

**Fig. 4:**
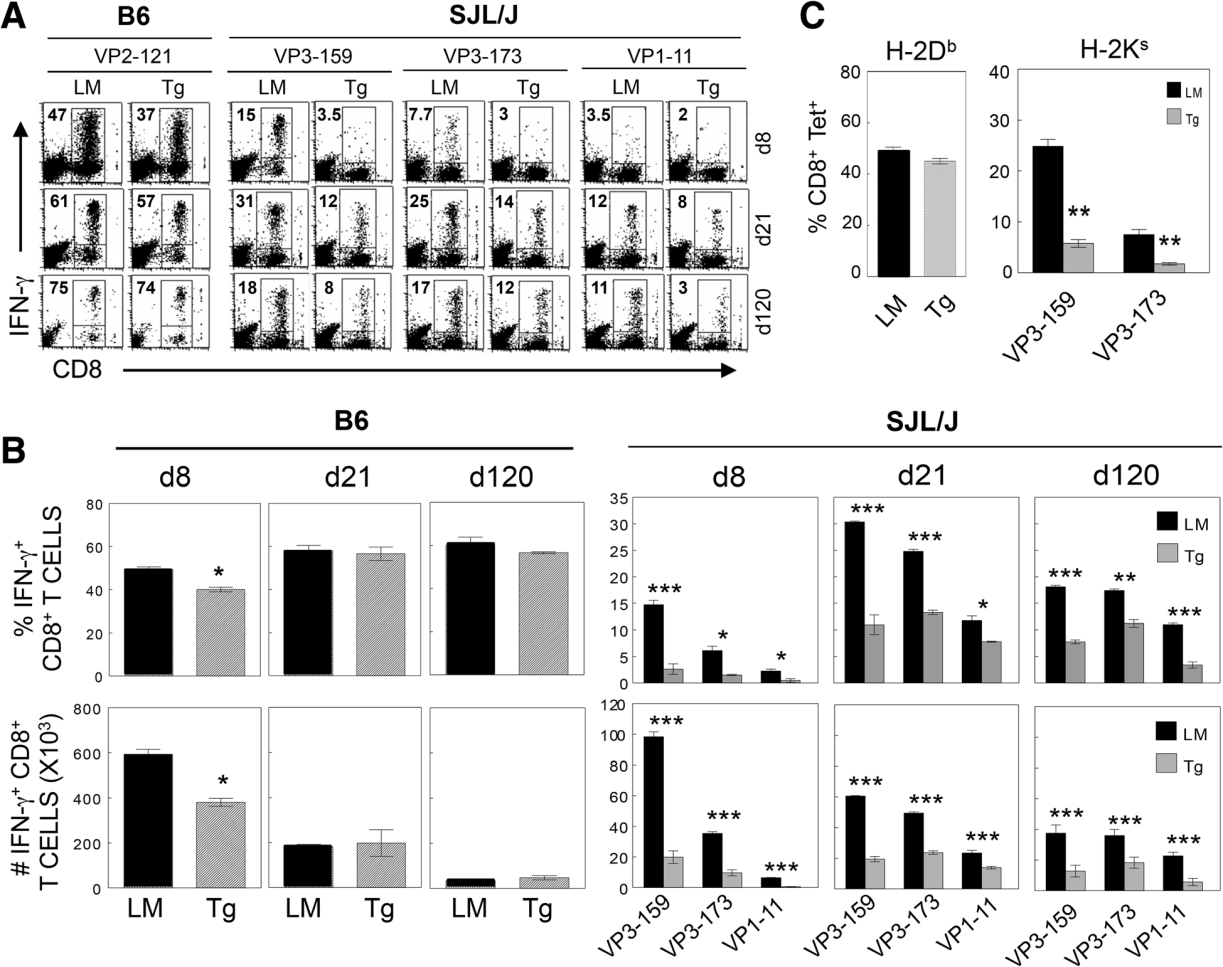
Antigen-specific IFN-γ-producing CD8^+^ T cell levels in the CNS of TMEV-infected P2/P3-Tg mice. **a** CNS-infiltrating cells from P2/P3-Tg mice and their littermates were isolated at 8, 21, and 120 days pi and stimulated with the indicated peptides (2 μM) for 6 h. Then, the cells were stained for both CD8 and intracellular IFN-γ, followed by flow cytometric analysis. The percentage of IFN-γ^+^ and CD8^+^ T cells is shown in the *upper left quadrant* of each plot. A representative flow cytometry plot from three independent experiments is shown. d8, d21, and d120 represent day 8, day 21, and day 120 post-TMEV infection, respectively. Tg and LM indicate P2/P3-Tg and littermate mice, respectively. **b** The proportions and the total numbers of epitope-specific IFN-γ-producing CD8^+^ T cells (*left* for B6 and *right* for SJL mice) are presented. **c** The proportions of D^b^-VP2_121–130_ tetramer-staining CD8^+^ T cells in the CNS of B6 mice and the proportions of K^s^-VP3-_159–166_ and VP3-_173–181_ tetramer-positive CD8^+^ T cells in the CNS of SJL mice at 8 dpi are shown. Values given represent the mean percentages or numbers (±SD) of the results from three independent experiments. *P < 0.05; **P < 0.01; and ***P < 0.001

To examine whether the above pan-tolerance of CD4^+^ T cell responses to TMEV in the P2/P3-Tg mice was restricted to CD4^+^ T cell responses, we assessed CD8^+^ T cell responses to viral epitopes (Fig. 4). The proportion (*P* = 0.04) and number (*P* = 0.016) of IFN-γ-producing CNS CD8^+^ T cells in response to the major VP2_121-130_ capsid epitope at 8 dpi were also significantly lower in the P2/P3-Tg B6 mice compared with their littermates, although the T cell responses became similar 21 days post-infection (Fig. 4a, b). The reduction in IFN-γ-producing TMEV capsid-specific CD8^+^ T cell responses in the P2/P3-Tg SJL mice was even more prominent and was sustained throughout the course of viral infection (Fig. 4a, b). The differences between the P2/P3-Tg SJL and control mice were significant [VP3_159–166_-specific CD8^+^ T cells (*P* = 0.002), VP3_173–181_-specific CD8^+^ T cells (*P* = 0.004), and VP1_11–20_-specific CD8^+^ T cells (*P* = 0.0009)]. Although IFN-γ production in response to CD8 epitopes was low, it was possible that the infected P2/P3-Tg mice mounted a comparable level of virus-reactive CD8^+^ T cells that did not produce IFN-γ. To test this possibility, we assessed the binding ability of CD8^+^ T cells to epitope-loaded MHC class I tetramers (Fig. 4c). Interestingly, VP2_121–130_-loaded H-2D^b^ molecules stained a comparable portion of CNS-infiltrating CD8^+^ T cells 8 dpi in the infected P2/P3-Tg B6 mice (Fig. 4c left). In contrast, the proportions of CD8^+^ T cells in the P2/P3-Tg SJL mice that bound to H-2K^s^ tetramers containing the VP3_159–166_ and VP3_173–181_ epitopes were significantly lower compared to the control SJL mice (Fig. 4c right), which is similar to the proportions of IFN-γ producing CD8^+^ T cells (Fig. 4a, b). These results suggest that the quality and quantity of virus-specific CD8^+^ T cells are more severely altered in P2/P3-Tg mice in the susceptible SJL background compared to P2/P3-Tg mice in the resistant B6 background.

### P2/P3-Tg mice display T cell unresponsiveness to viral epitopes in the periphery

**Fig. 5:**
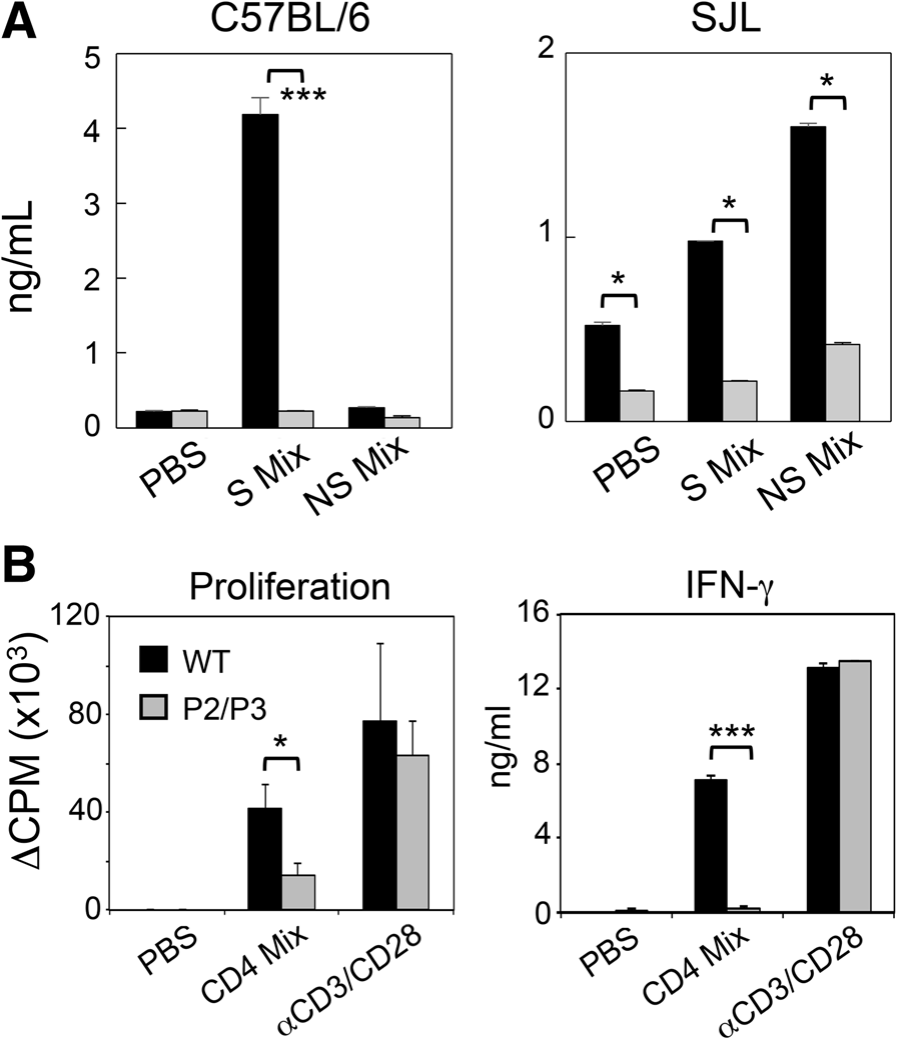
Effect of P2/P3 transgenes on the expression of cytokines in infected mice. **a** IFN-γ levels in the supernatants of the splenic cultures were determined by ELISA. Spleen cells were prepared 8 days post-infection from B6 and SJL mice (*n* = 3). Cells were cultured for 72 h in the presence of PBS or a mixture of 1 μM peptides. Culture supernatants were collected for ELISA. S mix for cells from B6 mice, VP2_206–220_, and VP4_25–38_; S mix for cells from SJL mice, VP1_233–250_, VP2_74–86_, and VP3_24–37_; and NS mix for both, 3D_6–23_ and 3D_20–38_. **b** The proliferative response of epitope-specific CD4^+^ T cells was measured based on [^3^H]-thymidine incorporation levels. Spleen cells from wild-type or P2/P3 transgenic B6 mice were stimulated with PBS, the CD4 peptide mix (VP2_206–220_ and VP4_25–38_) or plate-bound anti-CD3/CD28 antibodies. IFN-γ levels in the supernatants of the cultures were assessed with ELISA. Values given are the means (±SD) of the results from triplicate wells. *P < 0.05; and ***P < 0.001

Although T cell responses to viral epitopes in the CNS of infected P2/P3-Tg mice were significantly reduced (Figs. 3 and 4), it was unclear whether the reduced T cell responses were limited to the CNS or represented generalized T cell unresponsiveness to the virus. To address this issue, we analyzed virus-specific T cell responses in the spleen by measuring IFN-γ levels after stimulation with the indicated epitope peptides (Fig. 5a). Splenic T cells from infected P2/P3-Tg B6 mice produced drastically reduced levels of IFN-γ upon stimulation with P1-encoded capsid epitopes (S mix) compared to infected B6 mice. IFN-γ production in response to the P2/P3-encoded non-structural epitopes (NS mix) in the control mice was minimal. In contrast, IFN-γ levels produced by splenic T cells from P2/P3-Tg SJL mice in response to both the S mix and NS mix were significantly reduced compared to the control SJL mice. To confirm this virus-specific unresponsiveness, the proliferative response and IFN-γ production in response to viral epitopes versus general T cell stimulation (anti-CD3 and anti-CD28) were measured (Fig. 5b). The proliferative response of splenic T cells from P2/P3-Tg mice to viral epitope (CD4 mix) stimulation but not to pan T cell stimulation was significantly reduced compared to the control mice. Similarly, IFN-γ production by splenic T cells from the Tg mice in response to viral epitopes but not to pan T cell stimulation (anti-CD3/anti-CD28 antibodies) was also diminished. These results strongly suggest that P2/P3-Tg expression in mice induces T cell unresponsiveness to the entire viral epitopes including the transgene products but not to unrelated T cell responses.

We further tested the capacity of splenic CD11C^+^ dendritic cells and peritoneal macrophages from the P2/P3 Tg mice against purified splenic CD4^+^ T cells from TMEV-infected non-Tg mice (not shown). There were no differences in the efficiencies in the T cell stimulation between the antigen-presenting cells from the control vs. P2/P3-Tg mice. In addition, T cell responses of the P2/P3-Tg mice to unrelated ovalbumin antigen following ovalbumin immunization were not different from those of the control mice similarly immunized with ovalbumin (not shown). These results strongly suggested that the above reduction in the T cell responses is specific against viral determinants (Fig. 5) and no deficiencies in the T cell responses to unrelated antigens. In addition, the viability of T cells or antigen-presenting cells between control and the P2/P3-Tg mice was not different.

### Viral replication in vitro is compromised in various cell types from P2/P3-Tg mice

**Fig. 6:**
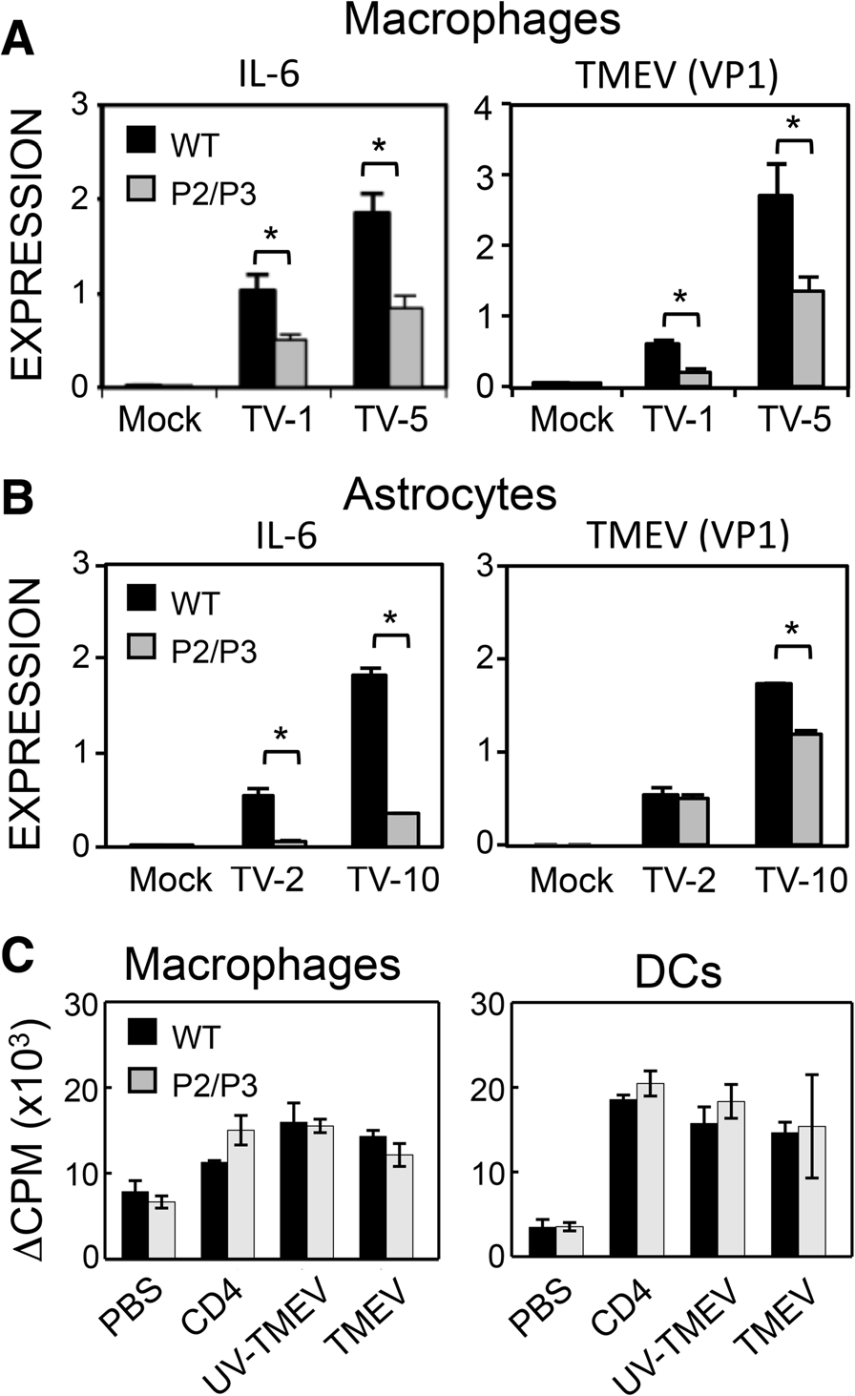
Expression of viral and IL-6 genes in different cell types. **a** IL-6 expression and virus replication were tested in peritoneal macrophages from P2/P3-B6 mice. Peritoneal macrophages were infected with 1 or 5 MOI of TMEV for 18 h. The level of TMEV-VP1 reflecting viral replication was compared by real-time PCR. **b** IL-6 expression and virus replication in astrocytes were also tested after infection with TMEV (MOI of 2 and 10) for 18 h. (**c**) Antigen-presenting function of cells from control and Tg mice was tested based on the induction of T cell proliferation. Peritoneal macrophages or splenic CD11C^+^ DC cells were prepared from WT and P2/P3 Tg B6 mice. Positively purified splenic CD4^+^ cells from TMEV-infected B6 mice were cultured with macrophages or DCs as APCs for 48 h in the presence of PBS, CD4 peptides, UV-TMEV, or infectious TMEV, followed by 18 h of incubation with 1 μCu ^3^H-TdR. Values given are the means (±SD) of the results from triplicate cultures. *P < 0.05

To elucidate the underlying mechanism of the reduced T cell responses in the P2/P3-Tg mice, we compared the levels of IL-6 (a representative innate pro-inflammatory cytokine) and viral replication between cells from P2/P3-Tg mice and the control mice. We tested two important cell types supporting viral persistence in the brain (macrophages and astrocytes) after 18 h of infection with TMEV in vitro. Both IL-6 production and virus replication were lower in the cells from the Tg mice (Fig. 6a, b), suggesting that the levels of viral replication and the associated pathogenic innate cytokines were compromised in cells expressing the P2/P3 transgenes.

Because the overall T cell responses toward TMEV were lower in the Tg mice compared to the WT mice (Figs. 3, 4, and 5), we compared the antigen-presenting function of virus-infected cells (Fig. 6c). To compare antigen presentation by virus-infected cells, we utilized CD11c^+^ cells and/or peritoneal macrophages isolated from the WT and Tg mice as APCs to stimulate virus-specific T cell proliferation. CD4^+^ T cells isolated from the spleens of TMEV-infected mice were co-cultured with the APCs in the presence of the CD4 peptide mix (already processed form), UV-TMEV (unprocessed form), and live TMEV (self-replicative form) for 72 h. Virus-specific T cell proliferation levels induced by the APCs did not differ between the APCs from the Tg and control mice (Fig. 6c), suggesting that the antigen-presenting function of APCs of the Tg mice was not affected.

### Replication of related viruses is inhibited in P2/P3-Tg mice

**Fig. 7:**
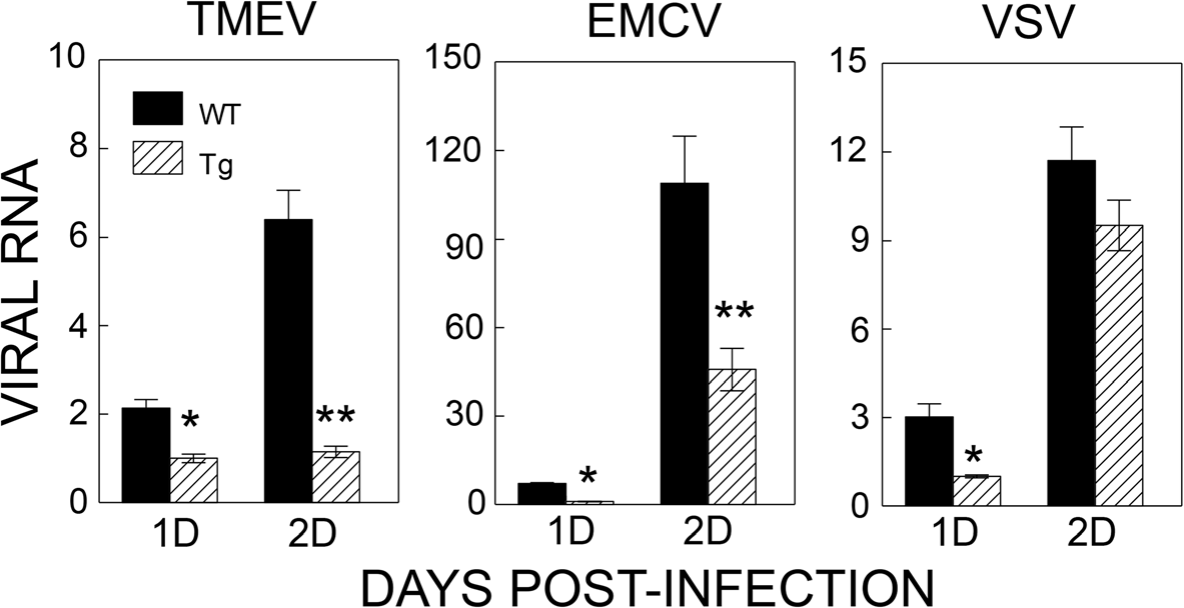
Effects of P2/P3 transgene expression on the replication of other viruses. Replication levels of TMEV, related EMCV, and unrelated VSV were tested after intracerebral infection of the viruses into control and Tg mice. Viral replication levels in the brains of infected mice 1 and 2 days post-infection were assessed using real-time PCR. Levels of viral genes were compared after normalization with GAPDH. Values given are the means (±SD) of the results from triplicate wells. *P < 0.05; **P < 0.01

We compared the replication of other viruses, including the closely related encephalomyocarditis virus (EMCV) and vascular stomatitis virus (VSV), in the P2/P3-Tg and control mice. We tested viral RNA levels 1 and 2 days after virus infection (Fig. 7). The mice died within 3 days of intracerebral infection of both VSV and EMCV due to severe viremia and wasting disease. Replication of both TMEV and the closely related EMCV were significantly reduced at both time points. However, the replication of unrelated VSV was transiently reduced (reduction was observed at day 1 but not day 2 post-infection). These results suggest that mice expressing the TMEV P2/P3 transgene display efficient inhibition of the replication of related viruses.

### In vitro expression of the P2 and P3 genes in NIH-3T3 cells inhibits viral replication

**Fig. 8:**
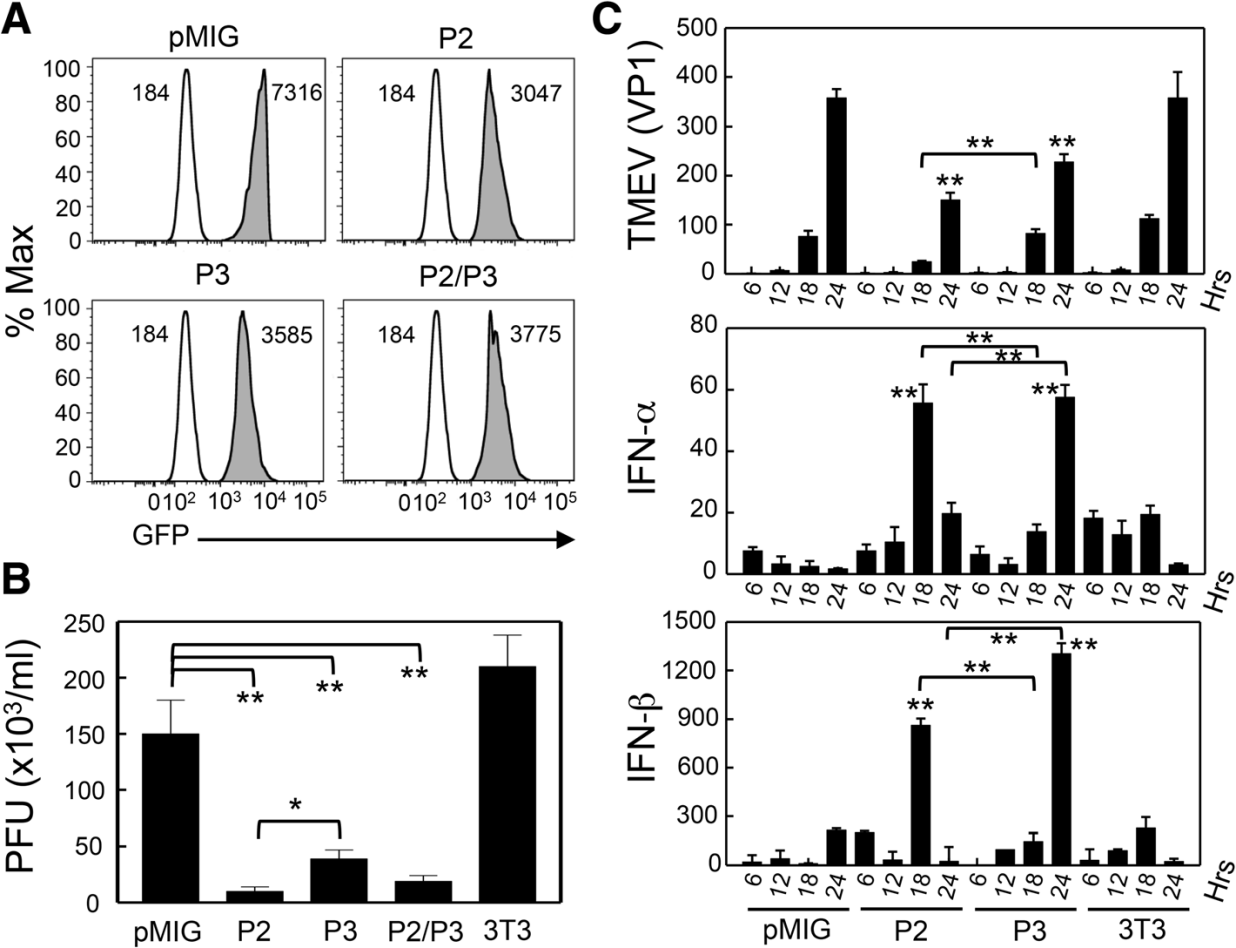
Decreased viral replication in NIH-3T3 cells transfected with the P2 and/or P3 genes. **a** Selection of NIH-3T3 cell lines expressing the P2, P3, and P2/P3 genes. Retroviruses containing the P2, P3, and P2/P3 genes were produced in Phoenix cells using the pMIG retroviral vector. NIH-3T3 cells were infected with the recombinant retroviruses. After a 2-week rest period, GFP-positive cells with MFI intensity between 3000 and 7500 were sorted with Moflo. Uninfected 3T3 cells (*clear histogram*) were compared with 3T3 cells infected with retroviruses containing the viral genes (*shaded histogram*). **b** Infectious virus levels produced 48 h post-infection with TMEV (MOI = 10) were compared by standard plaque assay using BHK-21. pMIG and 3T3 cells infected with retrovirus containing empty vector; P2, pMIG-P2; P3, pMIG-P3; P2/P3, pMIG-P2/P3; and 3T3, untransfected NIH-3T3 cells. **c** Expression of viral, IFN-α, and IFN-β messages was assessed by real-time PCR at 6, 12, 18, and 24 h post-infection. Values given are the means (±SD) of the results from triplicate wells. *P < 0.05; **P < 0.01

Because the P2/P3-Tg mice express both the TMEV P2 and P3 genes, it is unclear whether P2 or P3 is responsible for these inhibitory effects. Additionally, whether gene expression in a cell line can duplicate the inhibition of viral replication remains unknown. To assess these possibilities, we infected mouse NIH-3T3 cells with retroviruses carrying a GFP-containing vector (pMIG) alone or pMIG containing the P2, P3, or P2/P3 genes and sorted cells displaying similar GFP intensities (Fig. 8a). Viral loads were measured in these cells 6, 12, 18, and 24 h post-TMEV infection (Fig. 8b). Interestingly, 3T3 cells carrying P2, P3, or P2/P3 showed significantly reduced replication, with the most significant reduction observed in the P2-carrying cells. Viral RNA production levels in these cells (Fig. 8c) were consistent with the virion production measured by plaque assay (Fig. 8b). Interestingly, IFN-α and IFN-β levels in 3T3 cells carrying the P2 or P3 genes were significantly higher than either the uninfected or control (vector) retrovirus-infected cells (Fig. 8c). These results strongly suggest that the expression of the TMEV P2 or P3 gene induces elevated type I IFNs upon viral infection. Consequently, viral replication in cells expressing these genes is most likely inhibited via the down stream IFN-stimulating genes.

## Discussion

The expression of transgenes in mice has been utilized as an important tool to investigate various immune mechanisms, such as unresponsiveness, autoimmunity, and T cell development [18–20]. We previously demonstrated that transgenic mice carrying the TMEV P1 gene encoding a viral structural protein displayed reduced virus-specific immune responses, resulting in reduced pathogenesis in the transgenic mice despite the increased viral loads in the CNS [8]. Therefore, viral persistence does not always lead to TMEV-IDD, although viral persistence has long been believed to have a major impact on the development of TMEV-IDD [21].

In the present study, we established P2/P3-Tg mice to dissect the role of non-structural proteins in the development of TMEV-induced demyelinating disease. We demonstrated that P2/P3-Tg mice (particularly in the susceptible SJL background) displayed elevated viral persistence in the brain and later in the spinal cord (Fig. 2). However, P2/P3-Tg mice developed significantly less severe and less frequent disease (Fig. 2). These data suggest that viral persistence may not directly correlate with disease development. This finding is consistent with our previous data demonstrating that P1-Tg SJL/J mice with higher viral loads at all stages of viral infection compared to their littermates developed less severe disease [8]. The lack of correlation between viral persistence and disease development implies that viral persistence alone may not be able to drive the development of TMEV-IDD. Indeed, TMEV-infected P2/P3-Tg mice mounted lower levels of anti-TMEV T cell responses (Figs. 3, 4, and 5). The lower levels of protective antiviral T cell responses in the P2/P3-Tg mice under persistent inflammatory environment may have contributed to the elevated viral persistence in the CNS.

The P2/P3-Tg mice were expected to mount unaffected immune responses specific to the epitopes encoded by the P1 genes. However, the P2/P3-Tg SJL mice exhibited severely compromised virus-specific T cell responses to the structural epitopes encoded by the P1 region (Fig. 3b, c). The presence of excessive levels of type I IFNs is known to exert severe suppression of adaptive immune responses in SJL mice via the inhibition of development and function of antigen-presenting cells [22]. Therefore, the excessive production of type I IFNs in the cells expressing P2 and/or P3 genes, as well as P2/P3-Tg SJL mice (Figs. 6 and 8) most likely results in the compromised adaptive immune responses to viral antigens, which are responsible for the pathogenesis and the protection. Furthermore, the cells expressing the P2 and/or P3 genes produce lower levels of IL-6 upon TMEV infection (Fig. 6), which may curtail the generation of pathogenic T cells [23, 24]. Collectively, the lower initial viral replication leading to the low level of anti-viral T cell responses in P2/P3-Tg mice combined with the low level of pathogenic T cell responses may result in elevated viral persistence in the absence of the pathogenesis of demyelinating disease. In contrast to the viral persistence in the infected P2/P3-Tg SJL mice (Fig. 2), cells expressing the P2 and/or P3 genes from the P2/P3-Tg mice or cells transfected with P2 or P3 displayed reduced TMEV replication in vitro (Figs. 6 and 8). Because the viral replication experiments in vitro do not involve adaptive anti-viral immunity, the viral replication level in vitro does not correspond to viral persistence in the infected mice where strong adaptive immune responses are necessary to control viral persistence. Therefore, the protective type I IFNs that are elevated in the P2/P3-expressing cells may be sufficient to hinder viral replication in vitro.

Interestingly, the inhibition of viral replication is partially preferential for the same family of viruses because TMEV and EMCV, which belong to the same cardiovirus genus of the picornavirus family, are selectively inhibited compared to VSV, which is not a member of picornaviridae (Fig. 7). The underlying mechanism of viral replication inhibition is not clear. The elevated production of type I IFNs upon viral infection in cells from P2/P3-Tg mice (Fig. 8) suggests that these cytokine levels may affect viral replication. Recently, the expression of TMEV 3D in susceptible FVB mice showed an antiviral effect through elevated type I interferon expression [12]. Although the immune responses were not assessed in the TMEV DA strain 3D-Tg FVB mice, they concluded that adaptive immune responses are not an important contributing factor because 3D-expressing Rag-deficient FVB mice, which lack immune responses, provided protection. However, our results obtained with P2/P3-Tg mice (Figs. 3, 4, and 5) indicate that virus-specific adaptive immune responses also contribute to the pathogenesis of TMEV-induced demyelinating disease. Our attempts to individually express the 2A, 2B, 2C, 3A, 3C, and 3D genes to assess their inhibitory functions in the mouse cell line were not successful, suggesting that uncleaved non-structural proteins or contiguous viral genome sequences may be important for the inhibitory function (data not shown).

The protein encoded by the 3A gene in the P3 of poliovirus inhibits protein secretion by blocking endoplasmic reticulum-to-Golgi transport [25, 26]. This inhibition of protein secretion may play a role in the immune-evading mechanism of poliovirus through the down-regulation of MHC class I-dependent viral antigen presentation [27] and the reduction of innate cytokine secretion [28]. However, the inhibitory ability of the TMEV 3A protein was absent [29]. Interestingly, the uncleaved 2BC protein encoded by the P2 genes (rather than 3A) of foot-and-mouth disease virus (FMDV) exhibited a similar inhibitory function [30]. Therefore, 2BC of TMEV, which is similar to FMDV with respect to its resistance to bredfeldin A [30], may play an important role in immune evasion by dampening virus-specific T cell responses. In addition to P2, the expression of the P3 and P2/P3 genes also inhibited viral replication over twofold compared to the controls (Figs. 6 and 8). Therefore, a protein encoded by the genes or a viral genome segment of P3 appears to inhibit viral replication independently of P2. It is conceivable that the 3D region of TMEV in the P3 segment may inhibit viral replication as previously reported [12]. A very recent study using the TMEV DA 3D-Tg model has suggested that the catalytic site of 3D protein is associated with elevated production of type I IFNs [31]. Interestingly, cells expressing the P2 or P3 genes displayed elevated production of type I IFNs upon viral infection (Fig. 8). This result is consistent with the previous observation with 3D-Tg mice. However, our results with TMEV BeAn P2/P3-Tg mice suggest that the P2 region, in addition to the P3 (including 3D) is also capable of inducing the production of type I IFNs. Although we failed to detect any measurable levels of viral proteins encoded by the P2 and P3 genes based on the polyclonal rabbit antibodies to the primary amino acid sequences of the N-termini of 2A, 2B, and 3D, the potential involvement of the unfolded protein response (UPR) to the P2/P3 products, which is capable of inducing type I IFNs [32, 33], cannot be excluded. Nevertheless, distinct mechanisms must be operational between the P2 and P3 gene products in the induction of innate immune responses.

The underlying mechanism responsible for the elevated type I IFN production in cells expressing the P2 and/or P3 genes is not clear. It is conceivable that some host pattern-recognizing molecules (e.g., TLRs such as TLR7/8 and TLR9) recognize either the viral proteins or nucleic acids encoded by the P2/P3 region and consequently trigger innate cytokine production [34]. We previously showed that TMEV infection triggers TLR-mediated innate cytokine production in mice via interaction with viral double-stranded RNA (dsRNA) replication intermediates [35–37]. In addition, we demonstrated that TMEV infection triggers innate immunity in the host via melanoma differentiation-associated 5 (MDA-5) protein [38], which involves a group of intracellular responders, the retinoic acid-inducible gene I-like receptors (RLR) [39]. Signaling pathways of dsRNAs, non-self RNAs, and certain DNAs share the involvement of IPS-1/MAVS, which is associated with RLR-mediated signaling [40–42]. The protection against viral infection in the TMEV 3D-Tg model, which is dependent on the presence of MDA-5 or MAVS signaling [31], supports this possibility. Because viral DNA is transcribed in transgenic cells to produce viral RNAs encoding the P2 and P3 regions (Fig. 2), structural features of viral RNA branches in the P2 and P3 region of TMEV may be recognized by MDA-5 as previously proposed [43–45]. This possibility is supported by the fact that similar inhibition of viral replication is observed in cells containing the P2 and/or P3 regions as the RNA form after infection with the recombinant retroviruses (Fig. 8). This possibility is also consistent with the results indicating that cells expressing TMEV genes inhibit not only TMEV replication but also replication of closely related EMCV compared to that of unrelated VSV (Fig. 7). Nevertheless, viral DNA and/or RNA transcripts from the viral transgenes may trigger innate cytokine production via TLR-dependent and/or independent pathways [34, 46–48]. Further studies on the mechanism underlying the non-structural gene-mediated antiviral effect might lead to the development of powerful new antiviral drugs or treatments.

## Conclusions

We demonstrated here that transgenic SJL mice expressing the non-structural genes (P2 and/or P3) of TMEV showed severely suppressed anti-viral immune responses to broad viral determinants, including the epitopes encoded by the P1 structural genes. These P2/P3-Tg SJL mice were resistant to the TMEV-induced demyelinating disease despite their elevated viral loads in the CNS throughout the viral infection. However, the early viral loads (2–3 days) in the CNS of the P2/P3-Tg mice were low after infection with TMEV and closely related EMCV compared to unrelated VSV. Further experiments indicated that the production of type I IFNs in cells expressing the P2 and/or P3 RNAs was markedly elevated upon viral infection but the production of IL-6 was inhibited. Based on these results, we hypothesize that excessive production of type I IFNs in the Tg mice expressing the non-structural viral genes suppresses the induction of pathogenic as well as protective adaptive immune responses, resulting in the resistance to the demyelinating disease yet increased viral loads in the CNS. The fact that more efficient inhibition against a related virus compared to an unrelated virus strongly suggests a role of innate immunity induced by the P2 and P3 RNAs, which recognizes the similarity in the viral genome structures such as MDA-5. Alternatively, VSV may be able to respond to the innate immune responses differently from the related picornavirus family members. Nevertheless, the expression of viral non-structural genes may serve as a useful new method to prevent a broadly virus-specific pathogenesis in the hosts.

## Abbreviations

CNS, central nervous system; MS, multiple sclerosis; NS mix, peptide mix derived from non-structural proteins; PCR, polymerase chain reaction; PFU, plaque-forming unit; PI, post-infection; S mix, peptide mix derived from structural proteins; Tg, transgenic mice; TMEV, Theiler’s murine encephalomyelitis virus; TMEV-IDD, TMEV-induced demyelinating disease.

## References

[1] Dal Canto MC, Kim BS, Miller SD, Melvold RW (1996). Theiler’s murine encephalomyelitis virus (TMEV)-induced demyelination: a model for human multiple sclerosis. Methods.

[2] Kim BS, Lyman MA, Kang BS, Kang HK, Lee HG, Mohindru M, Palma JP (2001). Pathogenesis of virus-induced immune-mediated demyelination. Immunol Res.

[3] Lipton HL, Dal Canto MC (1976). Theiler’s virus-induced demyelination: prevention by immunosuppression. Science.

[4] Clatch RJ, Lipton HL, Miller SD (1987). Class II-restricted T cell responses in Theiler’s murine encephalomyelitis virus (TMEV)-induced demyelinating disease. II. Survey of host immune responses and central nervous system virus titers in inbred mouse strains. Microb Pathog.

[5] Yauch RL, Palma JP, Yahikozawa H, Koh CS, Kim BS (1998). Role of individual T-cell epitopes of Theiler’s virus in the pathogenesis of demyelination correlates with the ability to induce a Th1 response. J Virol.

[6] Kang BS, Lyman MA, Kim BS (2002). Differences in avidity and epitope recognition of CD8(+) T cells infiltrating the central nervous systems of SJL/J mice infected with BeAn and DA strains of Theiler's murine encephalomyelitis virus. J Virol.

[7] Mohindru M, Kang B, Kim BS (2006). Initial capsid-specific CD4(+) T cell responses protect against Theiler’s murine encephalomyelitis virus-induced demyelinating disease. Eur J Immunol.

[8] Myoung J, Bahk YY, Kang HS, Dal Canto MC, Kim BS (2008). Anticapsid immunity level, not viral persistence level, correlates with the progression of Theiler’s virus-induced demyelinating disease in viral P1-transgenic mice. J Virol.

[9] Jin YH, Kang B, Kim BS (2009). Theiler’s virus infection induces a predominant pathogenic CD4+ T cell response to RNA polymerase in susceptible SJL/J mice. J Virol.

[10] Lin X, Njenga MK, Johnson AJ, Pavelko KD, David CS, Pease LR, Rodriguez M (2002). Transgenic expression of Theiler’s murine encephalomyelitis virus genes in H-2^b^ mice inhibits resistance to virus-induced demyelination. J Virol.

[11] Kerkvliet J, Zoecklein L, Papke L, Denic A, Bieber AJ, Pease LR, David CS, Rodriguez M (2009). Transgenic expression of the 3D polymerase inhibits Theiler’s virus infection and demyelination. J Virol.

[12] Kerkvliet J, Papke L, Rodriguez M (2011). Antiviral effects of a transgenic RNA-dependent RNA polymerase. J Virol.

[13] Kang BS, Lyman MA, Kim BS (2002). The majority of infiltrating CD8+ T cells in the central nervous system of susceptible SJL/J mice infected with Theiler’s virus are virus specific and fully functional. J Virol.

[14] Pullen LC, Park SH, Miller SD, Dal Canto MC, Kim BS (1995). Treatment with bacterial LPS renders genetically resistant C57BL/6 mice susceptible to Theiler’s virus-induced demyelinating disease. J Immunol.

[15] Fuller AC, Kang B, Kang HK, Yahikozowa H, Dal Canto MC, Kim BS (2005). Gender bias in Theiler’s virus-induced demyelinating disease correlates with the level of antiviral immune responses. J Immunol.

[16] Jin YH, Mohindru M, Kang MH, Fuller AC, Kang B, Gallo D, Kim BS (2007). Differential virus replication, cytokine production, and antigen-presenting function by microglia from susceptible and resistant mice infected with Theiler’s virus. J Virol.

[17] Altman JD, Moss PA, Goulder PJ, Barouch DH, McHeyzer-Williams MG, Bell JI, McMichael AJ, Davis MM (1996). Phenotypic analysis of antigen-specific T lymphocytes. Science.

[18] Adams TE (1990). Tolerance to self-antigens in transgenic mice. Mol Biol Med.

[19] Hanahan D (1990). Transgenic mouse models of self-tolerance and autoreactivity by the immune system. Annu Rev Cell Biol.

[20] Pircher H, Rohrer UH, Moskophidis D, Zinkernagel RM, Hengartner H (1991). Lower receptor avidity required for thymic clonal deletion than for effector T-cell function. Nature.

[21] Tsunoda I, Fujinami RS (1996). Two models for multiple sclerosis: experimental allergic encephalomyelitis and Theiler’s murine encephalomyelitis virus. J Neuropathol Exp Neurol.

[22] Hou W, So EY, Kim BS (2007). Role of dendritic cells in differential susceptibility to viral demyelinating disease. PLoS Pathog.

[23] Hou W, Kang HS, Kim BS (2009). Th17 cells enhance viral persistence and inhibit T cell cytotoxicity in a model of chronic virus infection. J Exp Med.

[24] Hou W, Jin YH, Kang HS, Kim BS (2014). Interleukin-6 (IL-6) and IL-17 synergistically promote viral persistence by inhibiting cellular apoptosis and cytotoxic T cell function. J Virol.

[25] Doedens JR, Kirkegaard K (1995). Inhibition of cellular protein secretion by poliovirus proteins 2B and 3A. EMBO J.

[26] Doedens JR, Giddings TH, Kirkegaard K (1997). Inhibition of endoplasmic reticulum-to-Golgi traffic by poliovirus protein 3A: genetic and ultrastructural analysis. J Virol.

[27] Deitz SB, Dodd DA, Cooper S, Parham P, Kirkegaard K (2000). MHC I-dependent antigen presentation is inhibited by poliovirus protein 3A. Proc Natl Acad Sci U S A.

[28] Dodd DA, Giddings TH, Kirkegaard K (2001). Poliovirus 3A protein limits interleukin-6 (IL-6), IL-8, and beta interferon secretion during viral infection. J Virol.

[29] Choe SS, Dodd DA, Kirkegaard K (2005). Inhibition of cellular protein secretion by picornaviral 3A proteins. Virology.

[30] Moffat K, Howell G, Knox C, Belsham GJ, Monaghan P, Ryan MD, Wileman T (2005). Effects of foot-and-mouth disease virus nonstructural proteins on the structure and function of the early secretory pathway: 2BC but not 3A blocks endoplasmic reticulum-to-Golgi transport. J Virol.

[31] Painter MM, Morrison JH, Zoecklein LJ, Rinkoski TA, Watzlawik JO, Papke LM, Warrington AE, Bieber AJ, Matchett WE, Turkowski KL, Poeschla EM, Rodriguez M (2015). Antiviral protection via RdRP-mediated stable activation of innate immunity. PLoS Pathog.

[32] Ron D, Walter P (2007). Signal integration in the endoplasmic reticulum unfolded protein response. Nat Rev Mol Cell Biol.

[33] Ke PY, Chen SS (2011). Activation of the unfolded protein response and autophagy after hepatitis C virus infection suppresses innate antiviral immunity in vitro. J Clin Invest.

[34] Boehme KW, Compton T (2004). Innate sensing of viruses by toll-like receptors. J Virol.

[35] So EY, Kang MH, Kim BS (2006). Induction of chemokine and cytokine genes in astrocytes following infection with Theiler’s murine encephalomyelitis virus is mediated by the Toll-like receptor 3. Glia.

[36] So EY, Kim BS (2009). Theiler’s virus infection induces TLR3-dependent upregulation of TLR2 critical for proinflammatory cytokine production. Glia.

[37] Jin YH, Kang HS, Hou W, Meng L, Kim BS (2015). The level of viral infection of antigen-presenting cells correlates with the level of development of Theiler’s murine encephalomyelitis virus-induced demyelinating disease. J Virol.

[38] Jin YH, Kim SJ, So EY, Meng L, Colonna M, Kim BS (2012). Melanoma differentiation-associated gene 5 is critical for protection against Theiler’s virus-induced demyelinating disease. J Virol.

[39] Mogensen TH, Paludan SR (2005). Reading the viral signature by Toll-like receptors and other pattern recognition receptors. J Mol Med.

[40] Kawai T, Takahashi K, Sato S, Coban C, Kumar H, Kato H, Ishii KJ, Takeuchi O, Akira S (2005). IPS-1, an adaptor triggering RIG-I- and Mda5-mediated type I interferon induction. Nat Immunol.

[41] Seth RB, Sun L, Ea CK, Chen ZJ (2005). Identification and characterization of MAVS, a mitochondrial antiviral signaling protein that activates NF-kappaB and IRF 3. Cell.

[42] Ishii KJ, Akira S (2006). Innate immune recognition of, and regulation by, DNA. Trends Immunol.

[43] Pichlmair A, Schulz O, Tan CP, Rehwinkel J, Kato H, Takeuchi O, Akira S, Way M, Schiavo G, Reis e Sousa C (2009). Activation of MDA5 requires higher-order RNA structures generated during virus infection. J Virol.

[44] Luthra P, Sun D, Silverman RH, He B (2011). Activation of IFN-beta expression by a viral mRNA through RNase L and MDA5. Proc Natl Acad Sci U S A.

[45] Rodriguez KR, Bruns AM, Horvath CM (2014). MDA5 and LGP2: accomplices and antagonists of antiviral signal transduction. J Virol.

[46] Yu X, Tu C, Li H, Hu R, Chen C, Li Z, Zhang M, Yin Z (2001). DNA-mediated protection against classical swine fever virus. Vaccine.

[47] Zhu J, Martinez J, Huang X, Yang Y (2007). Innate immunity against vaccinia virus is mediated by TLR2 and requires TLR-independent production of IFN-beta. Blood.

[48] Pichlmair A (2007). Innate recognition of viruses. Immunity.

